# Necrology

**Published:** 1898-04

**Authors:** 


### NECROLOGY.

GEHEIMRAT RUDOLF LEUCKART.—We regret to announce the death of Geheimrat Rudolf Leuckart, Professor of Zoölogy and Comparative Anatomy in the University of Liepsig. Leuckart's name is too well known to veterinarians to need any commentary on his work. As the founder of the modern school of parasitology, and as the discoverer of many important facts in connection with the structure, life-history, and classification of animals, his name will be known as long as zoölogy is studied.

